# Long-term survival in Japanese renal transplant recipients with Alport syndrome: a retrospective study

**DOI:** 10.1186/s12882-018-1052-9

**Published:** 2018-10-03

**Authors:** Ai Katsuma, Yasuyuki Nakada, Izumi Yamamoto, Shigeru Horita, Miyuki Furusawa, Kohei Unagami, Haruki Katsumata, Masayoshi Okumi, Hideki Ishida, Takashi Yokoo, Kazunari Tanabe, Japan Academic Consortium of Kidney Transplantation (JACK)

**Affiliations:** 1 Division of Nephrology and Hypertension, Department of Internal Medicine, The Jikei University School of Medicine, 3-25-8, Nishi-Shimbashi, Minato-ku, Tokyo, 105-8461 Japan; 2 Department of Medicine, Kidney center, Tokyo Women’s Medical University, Tokyo, Japan; 3 Department of Urology, Tokyo Women’s Medical University, Tokyo, Japan

**Keywords:** Alport syndrome, Kidney transplantation, Type IV collagen

## Abstract

**Background:**

Patients with Alport syndrome (AS) develop progressive kidney dysfunction due to a hereditary type IV collagen deficiency. Survival of the kidney allograft in patients with AS is reportedly excellent because AS does not recur. However, several studies have implied that the type IV collagen in the GBM originates from podocytes recruited from the recipient’s bone marrow-derived cells, suggesting the possibility of AS recurrence. Limited data are available regarding AS recurrence and graft survival in the Japanese population; the vast majority were obtained from living related kidney transplantation (LRKTx).

**Methods:**

In this retrospective study, twenty-one patients with AS were compared with 41 matched patients without AS from 1984 to 2015 at two centers using propensity scores. Nineteen of the 21 patients with AS underwent LRKTx. The mean post-transplant follow-up period was 83 months in the AS group and 110 months in the control group. Histopathological AS recurrence was assessed by immunoreactivity of α5 (type IV collagen) antibody and electron microscopy.

**Results:**

The graft survival rate was equivalent between patients with and without AS (86.7% vs. 77.1% and 69.3% vs. 64.2% at 5 and 10 years; *p* = 0.16, log-rank test). Immunoreactivity to α5 antibody showed strong linear positivity with no focal defect in six patients. Electron microscopy showed no GBM abnormalities in two patients who were exhibiting long-term kidney allograft survival.

**Conclusions:**

We confirmed that α5 and the GBM structure were histopathologically maintained in the long term after kidney transplantation. The patient and graft survival rates were equivalent between Japanese patients with and without AS.

## Background

Alport syndrome (AS) is an inherited nephropathy characterized by sensorineural hearing loss and typical ocular abnormalities. It is caused by a hereditary type IV collagen deficiency [1–3]. In 85% of patients with AS, the collagen deficiency exhibits X-linked inheritance (COL4A5 gene), and the remaining 15% show autosomal recessive and rarely autosomal dominant inheritance (COL4A3 or COL4A4 genes). Histopathologically, these genetic alterations reflect glomerular basement membrane (GBM) thickening and lamellation, leading to focal segmental glomerulosclerosis and resultant global sclerosis and/or hyalinosis [1]. With respect to therapy, several studies have demonstrated the efficacy of angiotensin-converting enzyme inhibitors as the first-choice medications [4, 5], with angiotensin II receptor blockers and spironolactone as alternatives [6]. However, these medications are not always satisfactory, and there are no disease-specific medications. For these reasons, AS commonly progresses gradually from childhood, resulting in end-stage renal disease at a young age [3]. Indeed, according to the United States Renal Data System, the median age at which renal replacement therapy was initiated in AS from 2005 to 2009 was 33.7 years [7].

Patients with AS account for > 1% of all patients undergoing kidney transplantation [8]. Kidney allograft survival in patients with AS is excellent despite the fact that AS recipients can develop anti-GBM disease, which can induce allograft loss at a high rate early after kidney transplantation. These excellent results have been reported in several countries. These reasons were mainly explained by the lack of risk factors for chronic kidney disease (hypertension and diabetes mellitus), the absence of AS recurrence, and the very low rate of anti-GBM disease (only 1.9%) [9]. However, the clinical outcomes of renal allografts in the Japanese population, the vast majority of whom have undergone living related kidney transplantation (LRKTx), have not yet been investigated.

Another aim of this study was to evaluate the recurrence of AS in recipients of kidney transplantation. This topic was investigated for the following three reasons. First, the type IV collagen α3α4α5 heterotrimer in the GBM originates only from podocytes [10]. Second, there is evidence that allograft podocytes are produced from turnover of the recipient’s bone marrow-derived cells [11]. Third, experimental evidence has shown that the transplantation of wild-type bone marrow into irradiated Alport mice results in the recruitment of podocytes, which produce type IV collagen α5 within the damaged glomerulus [12, 13]. We hypothesized that recurrence of AS occurs locally and influences the clinical abnormalities of patients with AS.

To address these clinical and histopathological issues, we investigated the graft survival and histopathological changes in patients with AS as well as the expression of GBM type IV collagen α5 and electron microscopy findings in renal allograft recipients with AS in the Japanese population.

## Methods

We used the clinical, laboratory, and pathological data from recipients who underwent kidney transplantation at the Department of Urology, Tokyo Women’s Medical University, and the Division of Nephrology and Hypertension, Department of Internal Medicine, The Jikei University School of Medicine, from February 1984 to February 2015. Twenty-one recipients had AS as the primary renal disease leading to end-stage renal disease. Controls included patients who underwent transplantation with end-stage renal disease due to a condition other than AS. The two groups of patients were matched for recipient and donor age, number of renal transplants, presence of donor-specific antibodies, donor resources, and era of transplantation using propensity-score analysis. Endopoints were graft loss or patients death.

### Immunosuppressive regimens and desensitization protocols

All of the recipients were administered a triple immunosuppressive protocol comprising calcineurin inhibitors (CNI), antimetabolite drugs, and methylprednisolone (MP). Patients transplanted between 1989 and 1997 received cyclosporine and azathioprine (AZA), those transplanted between 1998 and 2000 received tacrolimus (TAC) and AZA, and those transplanted after 2001 received TAC and mycophenolate mofetil (MMF). After 2002, all patients received basiliximab perioperativelly. Splenectomy was performed at the time of transplantation between 1989 and 2004, and thereafter as an alternative to splenectomy, one dose of rituximab was administered 5–7 days before transplantation [14].

### Histopathology

Two independent observers performed histological evaluation of formalin-fixed paraffin sections stained with hematoxylin and eosin, periodic acid-Schiff, Masson’s trichrome, and periodic acid methenamine. All biopsies were evaluated based on the Banff 2013 classification.

### Immunofluorescence

Immunohistochemistry was performed on cold acetone-fixed frozen sections using fluorescein isothiocyanate-conjugated anti-α5 (IV) (H53 and B51) and Texas red-conjugated anti-α2 (IV) (H51) (Shigei Medical Research Institute, Okayama, Japan). The immunoreactivities of type IV collagen α2 and α5 were observed and photographed by fluorescence microscopy (DP-70; Olympus, Tokyo, Japan). To evaluate histological recurrence of AS, we selected all recipients who underwent allograft biopsy more than one year after kidney transplantation. We could not obtain frozen biopsy specimens from some of those patients because the biopsy performed long before. As a result, we could assess frozen specimens obtained from six recipients. We evaluated two to three glomeruli per patient to identify focal or diffuse defects of type IV collagen α5 immunoreactivities in the GBM.

### Electron microscopy

We observed the electron microscopic structure of the GBM in six patients of whom we could survey the allograft specimens in the long term more than one year after transplantation (Case1, 11, 12, 15, 16, 19). Among them we evaluated the electron microscopic figure of the GBM in two patients of whom we could survey the allograft specimens in particularly long term (101 and 110 months after kidney transplantation). For each, a total of 10 GBM measurements were evaluated on 10 randomly selected glomerular capillaries to determine the average (arithmetic mean) GBM thickness. Each electron micrograph was reviewed to determine whether foci of splitting or lamellation of the GBM was present.

### Biopsy and definition of AS recurrence

Graft biopsy was performed based on episodic hematuria and proteinuria or worsening renal function. The other protocol biopsies were performed at 3 months, 1 year, and 3 years after kidney transplantation. We defined the recurrence of AS as either focal or diffuse defects of immunoreactivities of type IV collagen α5 chains in the GBM. On electron microscopy, we defined the recurrence of AS as the significant progression of irregular thinning and thickening of the GBM with splitting and lamellation of the lamina densa.

### Statistical analyses

The baseline characteristics are presented as median with range or mean with standard deviation. We used Fisher’s exact test to compare categorical variables between groups and the Mann–Whitney test to compare continuous variables. Allograft and patient survival were evaluated using the Kaplan–Meier method and compared between groups using the log-rank test. A two-tailed *p*-value of < 0.05 was considered statistically significant.

## Results

### Patient characteristics

The characteristics of patients with AS and without AS are listed in Table 1. None of the variables except donor sex was significantly different between the two groups. The median age of the recipients was not significantly different between the AS and control groups [22 (range, 15–56) and 23 (range, 9–71) years, respectively; *p* = 0.94]. The median follow-up period was 99.5 months (range, 0–348 months) and was not significantly different between the two groups (*p* = 0.78). A total of 71.4% (15/21) of the patients in the AS group and 65.9% (27/41) in the control group were male. Most patients underwent their first kidney transplantation (AS vs. control, 95.2% vs. 97.5%, respectively), and the rates of LRKTx were not significantly different between the two groups (90.5% in AS group vs. 92.7% in control group, *p* = 1.0). In our institutes, LRKTx occupy large majority of kidney transplantation. Among them, a few deceased donor kidney transplantation (DDKTx) were performed, which were 2/21(9.5%) and 3/41(7.3%) in each Alport group and control group. Average age of recipient and donor were 29/20 and 53/28 respectively in each group (Alport group/control group). In DDKTx cases, number of renal transplants, presence of donor-specific antibodies, and era of transplantation were comparable in both groups, without any significant difference. Human leukocyte antigen mismatch, ABO compatibility, preformed donor-specific antibody, and the era of kidney transplantation were not significantly different between the two groups. The primary disease in 10 patients (24.4%) in the control group (*N* = 41) was predominantly reflux nephropathy, followed by IgA nephropathy in 5 patients (12.2%), focal segmental glomerulosclerosis in 3 (7.3%), diabetic nephropathy in 1 (2.4%), other conditions in 12 (29.3%), and unknown in 6 (14.6%) (Table 2). Fifteen of 21 recipients were male, and the donors comprised 10 fathers, 3 mothers, and 2 cadavers in AS group. Six female recipients underwent donations from three fathers, two mothers, and one husband (Table 3). We could obtain the information of hematuria in 43 recipients (69.3%, Alport group: 18cases, matched control group: 25cases) at the latest urine sample. Only few patients showed positive hematuria at the latest urine examination. There was no statistical difference in patients who showed positive hematuria between Alport group (2/18: 11.1%) and matched control group (5/25: 20.0%). We investigated the episodes of rejection in Alport group and control group. The rate of cases who underwent rejection were 8/17 (47.1%), 20/33 (60.6%) in Alport group and Control group, respectively. The results were not significantly different in both groups.

**Table 1:** Baseline characteristics in AS and control groups

Variables	Alport group	Matched Control group	*p* value
N	21	41	
Recipient age	22 (15–56)	23 (9–71)	0.94
Recipient gender (Male)	15 (71.4%)	27 (65.9%)	0.78
Recipient BMI	18.3 (14.4–22.9)	20.2 (14.6–27.2)	0.083
Time on Dialysis (month)	12 (0–193)	25 (0–137)	0.070
RRT modality (HD)	17 (81.0%)	33 (80.5%)	1.0
Donor age	52 (40–65)	50 (16–78)	0.42
Donor gender (Male)	15 (71.4%)	15 (37.5%)	0.015*
No. of Transplantation			
first	20 (95.2%)	40 (97.5%)	1.00
second	1 (4.8%)	1 (2.4%)	NA
third	0 (0.0%)	0 (0.0%)	
HLA-mismatch			
Class I	1.33 ± 0.73	1.46 ± 0.71	0.46
Class II	0.62 ± 0.59	0.78 ± 0.42	0.17
ABO incompatible	5 (23.8%)	9 (22.0%)	1.0
Graft weight	170 (125–280)	175 (120–270)	0.50
WIT (min)	5 (0–15)	5 (0–24)	0.67
TIT (min)	73 (39–660)	67.5 (44–2233)	0.55
Preformed DSA^a^			
HLA-Class I	0/9 (0.0%)	5/21 (23.8%)	0.30
HLA-Class II	0/6 (0.0%)	3/19 (15.8%)	1.0
Graft failure	6 (28.6%)	19 (45.2%)	0.27
Chronic rejection	4	14	0.89
Non-compliance	1	1	0.073
Unknown	1	4	NA
Follow up period (month)	83 (5–315)	110 (0–348)	0.78
Living donor RTx	19 (90.5%)	38 (92.7%)	1.0
Era of transplantation			
-1989	3 (14.3%)	5 (12.2%)	1.0
1990–1999	4 (19.0%)	11 (26.8%)	0.55
2000–2009	6 (28.5%)	18 (43.9%)	0.28
2010-	8 (38.1%)	7 (17.1%)	0.12
Hematuria^b^	2/18 (11.1%)	5/25 (20.0%)	0.69

DSA: donor-specific antibody, RTx: renal transplantation, TCMR: T cell-mediated rejection, ABMR: Antibody-mediated rejection

^a^Preformed DSA was evaluated by HLA-antibody single antigen test (Luminex method). This test for HLA-Class I was performed to nine cases in AS and twenty-one cases in control, and for HLA-Class II to six cases in AS and nineteen cases in control

^b^Hematuria displays the number of recipients who had hematuria more than (1+) at the latest examination/total recipients who could be obtained the information of latest urine examination

*p-value was < 0.05

**Table 2:** Primary disease in control group

Primary disease	Number (%)
Reflux nephropathy	10 (24.4%)
IgA nephropathy	5 (12.2%)
FSGS	3 (7.3%)
Hypoplastic kidney	2 (4.9%)
RPGN	2 (4.9%)
Diabetic nephropathy	1 (2.4%)
Others	12 (29.3%)
Unknown	6 (14.6%)

**Table 3:** Twenty-one kidney transplantation recipients with Alport syndrome

Case No.	Living/Cadaver	ABO-compatibility	Recipient	Donor		f/u period (month)	UP^a^	Hematuria^b^	Latest creatinine in serum	Recurrence of AS^c^	Graft loss	Causes of graft loss
			Age	Type/Relation	Age							
1	Living	Incompatible	26–30	Father	56–60	36	(+)	(−)	Loss	(−)	(+)	CR
2	Living	Compatible	26–30	Father	46–50	131	(−)	(−)	1.59		(−)	
3	Living	Incompatible	16–20	Father	41–45	5	(−)	unknown	1.16		(−)	
4	Living	Compatible	56–60	Husband	56–60	12	(−)	(−)	0.78		(−)	
5	Living	Incompatible	16–20	Mother	46–50	110	(−)	unknown	Loss		(+)	CR
6	Living	Compatible	26–30	Mother	56–60	5	(−)	(+)	1.06		(−)	
7	Living	Compatible	21–25	Father	46–50	290	(+)	(−)	Loss		(+)	CR
8	Living	Compatible	26–30	Father	56–60	276	(−)	(−)	1.95		(−)	
9	Living	Compatible	21–25	Father	56–60	239	(+)	(−)	1.62		(−)	
10	Living	Compatible	16–20	Father	46–50	216	(−)	unknown	1.4		(−)	
11	Living	Compatible	21–25	Father	56–60	180	(−)	(−)	2.17	(−)	(−)	
12	Living	Compatible	22–25	Father	56–60	120	(+)	(−)	Loss	(−) TBMD	(+)	CR
13	Living	Compatible	31–35	Father	56–60	83	(−)	(−)	1.31		(−)	
14	Living	Incompatible	21–25	Father	51–55	47	(−)	(−)	1.32		(−)	
15	Living	Incompatible	21–25	Father	46–50	24	(−)	(−)	1.16	(−)	(−)	
16	Living	Compatible	21–25	Father	46–50	11	(−)	(−)	1.44	(−) TBMD	(−)	
17	Living	Compatible	16–20	Mother	51–55	204	(−)	(−)	1.35		(−)	
18	Living	Compatible	21–25	Mother	46–50	39	(+)	(−)	Loss		(+)	NC
19	Living	Compatible	21–25	Mother	46–50	56	(−)	(+)	1.58	(−)	(−)	
20	Cadaver	Compatible	16–20	Other	36–40	315	(+)	(−)	Loss		(+)	unknown
21	Cadaver	Compatible	41–45	Other	61–65	35	(−)	(−)	1.92		(−)	

*KTx* kidney transplantation, *Bx* allograft biopsy, *AS* Alport syndrome, *N.D.* no data, *f/u* follow up, *UP* urine proteinuria, *NC* non-compliance, *CR* chronic rejection, *TBMD* thin basement membrane disease. ^a^Positive of urine proteinuria was defined as more than one plus by urinary qualitative examination. ^b^Positive hematuria was defined as more than one plus at the latest examination. ^c^Recurrence of AS was defined as no appearance of a deficiency of collagen α5 in glomerular basement membrane

### Patients and graft survival in AS and matched control groups

**Fig. 1:**
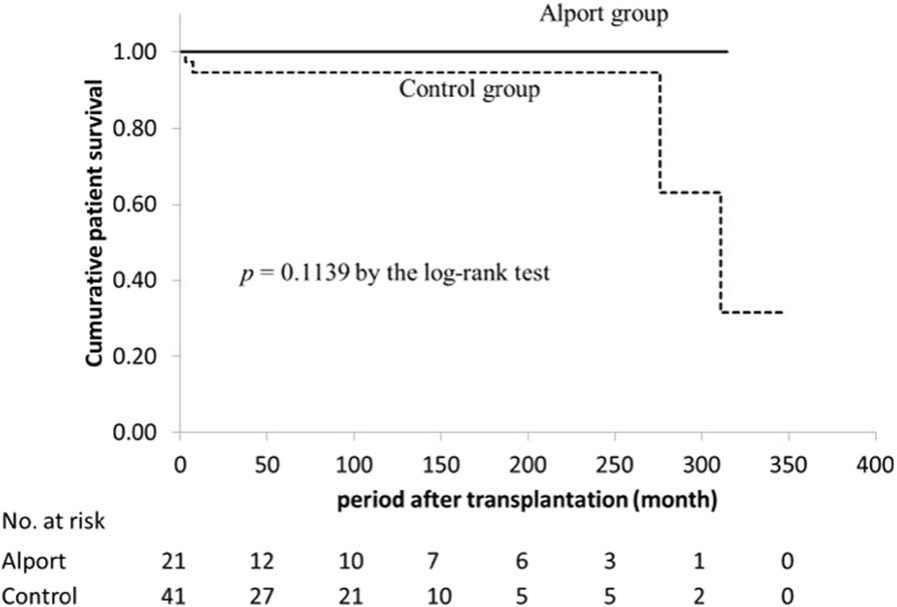
Cumulative patient survival comparison between AS group and matched control group

**Fig. 2:**
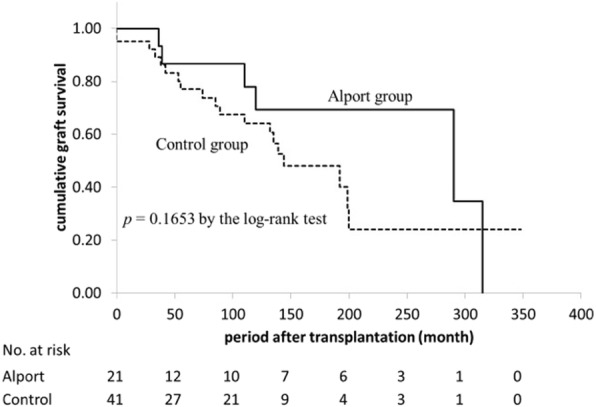
Cumulative graft survival (death-censored) comparison between AS group and matched control group

The cumulative patient survival rate was not significantly different between the two groups (*p* = 0.11, log-rank test) (Fig. 1). The 10-year patient survival rate after transplantation was 100% in the AS group and 94.6% in the matched control group. The cumulative graft survival rate was higher in the AS group (100%, 86.7%, and 69.3% at 1, 5, and 10 years, respectively) than that in the control group (95.1%, 77.1%, and 64.2% at 1, 5, and 10 years, respectively), but the difference was not significant (*p* = 0.16, log-rank test) (Fig 2). Five patients (28.6%) in the AS group and 19 (45.2%) in the matched control group developed allograft failure mainly caused by chronic rejection (66.7% in AS group vs. 73.7% in control group) or by non-compliance (16.0% in AS group vs. 5.3% in control group). There were no cases of recurrent nephropathy in the AS group.

### Long-term immunoreactivity of type IV collagen in GBM of AS allograft specimens after kidney transplantation

**Fig. 3:**
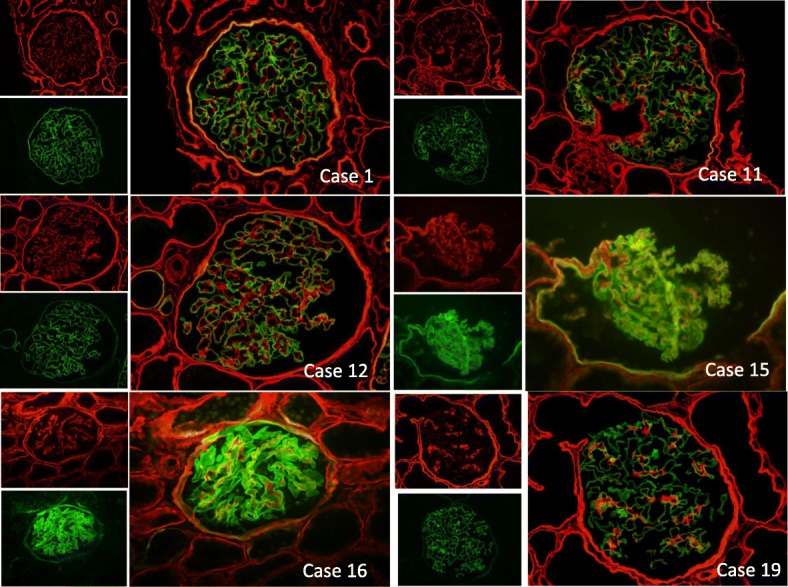
Immunofluorescence staining of type IV collagen α5 chain. Immunofluorescence staining of type IV collagen α2 chain (red), α5 chain (green), and merged images (combined red and green) of GBM on allograft biopsy specimens of AS recipients performed more than 1 year after transplantation (patient characteristics are shown in Table 3). In all cases, the type IV collagen α5 chain was stained linearly in the GBM without defects. Case1: A 27-year-old woman. Allograft biopsy specimen at 33 months after transplantation. The pathological diagnosis was chronic active antibody-mediated rejection. Case 11: A 21-year-old man. Allograft biopsy specimen at 101 months after transplantation. The pathological diagnosis was arteriolar hyalinosis. Case 12: A 21-year-old man. Allograft biopsy specimen at 110 months after transplantation. The pathological diagnoses were chronic active antibody-mediated rejection and IF/TA, moderate. The immunoreactivity of type IV collagen α2 chain was slightly increased in the mesangial and subendothelial regions compared with that in the other cases. Case 15: A 26-year-old man. Allograft biopsy specimen at 12 months after transplantation. The pathological diagnosis was minimally aggressive tubulointerstitial rejection, mild. Case 16: A 26-year-old man. Allograft biopsy specimen at 12 months after transplantation. The pathological diagnosis was no evidence of rejection. Case 19: A 23-year-old man. Allograft biopsy specimen at 14 months after transplantation. The pathological diagnosis was IF/TA, mild

We evaluated the graft specimens in 6 of 21 patients in the AS group who underwent allograft biopsy more than 1 year after kidney transplantation (Table 3). The median age of these patients at the time of kidney transplantation was 24 years (range, 21–27 years), and five of the six patients were male. The median duration of time from kidney transplantation to allograft biopsy was 23.5 months (range, 12–110 months). Type IV collagen α5 chains in the GBM of all patients were maintained linearly, and even focal depletion was not observed (Fig. 3). In one patient with chronic active antibody-mediated rejection, the α2 chains showed thickening in the GBM that extended to subendothelial lesions (Fig. 3, Case 12).

### Long-term electron microscopic findings of GBM in allograft specimens of AS recipients after kidney transplantation

We assessed the electron microscopic findings of six patients who underwent allograft biopsy. Cases 11 and 16 (data not shown) showed a focally thin basement membrane in the GBM, suggesting the transmission of thin basement membrane disease. Among these patients, we evaluated detailed long-term data after kidney transplantation (101 and 110 months after transplantation in Cases 11 and 12, respectively). We found no significant changes, such as splitting or lamination of the GBM, in both cases. Case 11 showed a thickness of 275 nm at 101 months after kidney transplantation 339 nm at 0 h. Case 12 showed a thickness of 220 nm at 110 months after transplantation and 146 nm at 0 h, suggesting the transmission of thin basement membrane disease. This case was characterized by chronic antibody-mediated rejection by light microscopy and showed a double-countered GBM with extended edema of the subendothelial lesion and fusion of foot process, suggesting endothelial and podocyte injury.

## Discussion

This study is the first to demonstrate patient and graft survival in the Japanese population of patients with AS. We retrospectively reviewed the long-term outcomes of 21 patients with AS who underwent kidney transplantation with a mean follow-up duration of 83 months and found that the cumulative patient and graft survival rates in the AS group were not significantly different from those of a well-matched control group (Figs. 1 and 2). Our data as well as those reported by Yilmaz from Turkey showed no difference between patients with and without AS [15]. In contrast, Temme showed better graft survival (HR, 0.75; 95% CI, 0.60–0.93; *p* = 0.008) and patient survival (HR, 0.46; 95% CI, 0.30–0.59; *p* = 0.001) in patients with AS using the ERA-EDTA Registry data [7]. The only difference in the patients’ characteristics was the rate of deceased donors (63.0% in the ERA-EDTA data, 32.0% in Yilma’s data, and 9.5% in our data). Notably, the author demonstrated that the patient and graft survival rates were better in deceased donors than in controls, but not in living donors. This finding suggests that living donors with AS could negatively affect patient and graft survival. In line with this, Gross from Germany observed six cases of living donor kidney transplantation in AS from relatives with mild urinary abnormalities and found that three of the six donors developed new-onset hypertension and two of the six donors developed new-onset proteinuria. The author pointed out that, genetically, AS may also affect other family members, and physicians should be aware of an increased risk of renal failure in both the donor and the recipient [16]. In this context, in countries with a donor shortage, such as Japan (in which almost 90% of transplantations are living donor kidney transplantations), careful evaluation and monitoring of both donors and recipients with AS are necessary. In the present study, 13 of 21 patients received kidneys from a father, suggesting that most donations were from an unaffected father with X-linked (COL4A5 gene) AS. However, we found at least two patients (Cases 12 and 16) with a relatively thin GBM on electron microscopy, suggesting possible transmission of thin basement membrane disease from autosomal-recessive (COL4A3 or COL4A4 gene) affected relatives [17, 18]. For example, in Case 16, we confirmed that type IV collagen α5 staining of the recipient’s native kidney biopsy revealed positive in the basement membrane of Bowman’s capsule but negative in GBM, suggesting autosomal-recessive AS associated with COL4A3/4 gene abnormality. Of note, previous report showed that about 40% of TBMD patients have heterozygous mutations at the COL4A3/4 locus [19]. Therefore, the donor in Case 16 could be heterozygous carrier with autosomal-recessive AS, resulting in TBMD shown in baseline allograft biopsy. These two patients had stable kidney function and did not show proteinuria or hematuria. These results suggest that living donor kidney transplantation from relatives with AS is acceptable if the donors and recipients are carefully evaluated. Another important factor associated with patient and graft outcomes is the occurrence of anti-GBM disease. Anti-GBM disease induces graft failure in the early stage of kidney transplantation in recipients with AS, but the incidence is generally very low at 1.9% [9]. Fortunately, no recipients in the AS group developed anti-GBM disease within the observation. Among patients with AS, the risk factors associated with graft loss in patients other than living donors and those with anti-GBM disease have not been fully evaluated. In the present study, 6 of 21 (28.6%) recipients with AS developed graft loss. Of these, four (66.6%) were due to chronic rejection, one (16.7%) was due to noncompliance with medication, and another was of unknown cause. These results are comparable with the causes of graft loss in the matched control group.

Another main finding of this study is that no patients developed recurrence of AS. To the best of our knowledge, this is the first study to evaluate the recurrence of AS in detail using histopathological methods. Generally, recurrent glomerulonephritis or glomerulopathy may cause graft loss in kidney transplantation, but most nephrologists believe that patients with AS do not develop recurrence. However, several recent clinical and experimental studies have implied that GBM type IV collagen originates from podocytes recruited from the recipient’s bone marrow-derived cells, suggesting the possibility of recurrence of AS. For example, Abrahamson clearly demonstrated that α3α4α5 chains are produced only by podocytes, as evidenced by immunoelectron microscopic examination [10]. In human kidney transplantation research, Becker showed that recipient-derived podocytes were found in 4 of 8 biopsies, representing 3 of 6 patients, and that 5 of the 740 podocytes examined in the female-donated allograft were male-derived, using fluorescence in situ hybridization for the X and Y chromosomes [11]. In addition, Sugimoto et al. reported that bone marrow transplantation from wild-type mice to Alport model mice (COL4A3 −/−) improved the symptoms of AS and the level of protein produced from bone marrow-derived cells [12]. Therefore, we initially hypothesized that the podocytes of allografts can be replaced by recipient-derived podocytes that cannot synthesize α3α4α5 chains, resulting in deficiency of the GBM of allograft. However, we found that the structure of the GBM remained normal and that no deficiencies of the α5 chains in the GBM occurred, even long after kidney transplantation, suggesting that AS does not recur histologically. We speculated following two mechanisms for this phenomenon:1) a very small number of donor derived podocytes were replaced by recipient derived ones [11], or 2) podocytes injury is rare in recipients with AS, and both podocytes and GBM turnover was not evident [20–22]. Additionally, only few recipients in Alport group showed hematuria in the latest examination (11.1%, Table 1), which further strengthen no histological recurrence of AS because electron microscopy might underestimate the presence of GBM changes of AS due to sampling bias.

The strengths of this study include the selection of controls (propensity-score analysis with matching of six factors: recipient and donor age, number of transplants, donor-specific antibody positivity, donor resources, and era of transplantation) and longer observational periods than in previous reports (99.5 vs. 75.4 months) [15]. Limitations include the retrospective design, small sample size due to the rarity of AS, lack of genetic information, and performance of few allograft biopsies because the allograft function was relatively good, even long after kidney transplantation, in recipients with AS.

## Conclusion

Our data demonstrate that graft survival in patients with AS is similar to that in patients with other primary diseases in the Japanese population. Careful selection of living donors is necessary for the successful management of kidney transplantation in patients with AS. Neither deficiency of the α5 chains (type IV collagen) nor GBM abnormalities were detected in the kidney allograft biopsies, indicating that AS recurrence was not present or was very limited in recipients with AS. Further pathological analysis with a longer follow-up to evaluate the possible recurrence of AS and transmission of thin basement membrane disease from affected relatives will contribute to a better understanding of kidney transplantation in patients with AS.

## Abbreviations

ABMR: antibody-mediated rejection
AS: Alport syndrome
DDKTx: deceased donor kidney transplantation
GBM: glomerular basement membrane
IF/TA: interstitial fibrosis and tubular atrophy
KTx: kidney transplantation
LRKTx: living related kidney transplantation
